# Principles of surgical treatment of Zenker diverticulum

**Published:** 2012-03-05

**Authors:** A Constantin, IN Mates, D Predescu, P Hoara, FI Achim, S Constantinoiu

**Affiliations:** * “Carol Davila” University of Medicine and Pharmacy, Bucharest, Romania; ** General and Esophageal Surgery Clinic, “Sf. Maria” Clinic Hospital, Bucharest, Romania

**Keywords:** Zenker, esophageal diverticulum, diverticulectomy, diverticulopexy

## Abstract

**Background:** Pharyngo-esophageal diverticula are most frequently described in elderly patients, having symptoms such as dysphagia, regurgitation, chronic cough, aspiration and weight loss. The etiology remains controversial, although most of the theories are linked to structural or functional abnormalities of the crico-pharyngeal muscle. With the therapeutic attitude varying from conservative to surgical (with associated morbidity and mortality), the importance of knowing the etiopathology and clinical implications of the disease for establishing the management of the case is mandatory. The aim of the study is the reevaluation of the methods and therapeutic principles in pharyngo-esophageal diverticular disease, starting from the etio pathogeny.

**Materials and Methods:** Our study group is made up of 11 patients with surgical indication for Zenker diverticulum, operated between 2001 and 2011.

**Results:** During that period, more patients were diagnosed with this pathology, but the surgical indication was carefully established, in conformity with the actual practice guides, which involve the evaluation of the clinical manifestations determined by the diverticulum, as well as the identification and interception of the pathological mechanisms by the therapeutic gesture.

**Conclusion:** Although it has a “benign” pathology, the esophageal diverticulum requires complex surgical procedure that implies significant morbidity.

**Abbreviations** UES= upper esophageal sphincter; NPO= nothing by mouth

## Introduction

Zenker diverticulum is the most common proof of a primitive pharyngo-esophageal motility disorder. The exact etiology remains unclear; the most accepted theory is that the disease is consequent to a functional disorder of pharyngo-esophageal motility, represented by increased resting pressure of the upper esophageal sphincter, lack of relaxation during swallowing and especially the lack of synchronization between upper esophageal sphincter and hypopharynx during swallowing. 

Ellis et al. supports the idea that relaxation and early closing of the upper esophageal sphincter (UES) during swallowing is the main cause of developing Zenker diverticulum [**1**]. Using manometry combined with video radiography, Cook et al. have not found the lack of synchronization between the motor activity of hypopharynx and UES during swallowing, but manometry showed inadequate relaxation with the rise of intraluminal pressure. More than that, histology studies proved degenerative modifications of the muscular layer, supporting inadequate relaxation by the lack of muscular elasticity at this level [**2**].

The degeneration of the striate muscular fibers of crico-pharyngeal muscle was seen at the histological examination; which are gradually replaced by fibro-fatty tissue [**3,4**]. This process (demonstrated at immuno-histochemistry), also affects the muscularis propria layer of the cervical esophagus [**5,6**]. However, these data were not confirmed by other similar communications [**7**].

Regardless of the type and cause of the pharyngo-esophageal motor abnormalities, most of the authors agree that the existence of the diverticulum at this level is the consequence of the intraluminal rise of the pressure, related to the resistance of the esophageal wall. 

Exceptional, pharyngo-esophageal diverticulum can be a rare complication after the surgery of the cervical spine trauma (dislocation, fracture), that uses metal plate for fixation. The trauma of the pharyngeal posterior wall occurs intraoperatively and can pass unnoticed. This leads to the appearance of a weak area, with the possibility of developing a pharyngo-esophageal diverticulum for the next years after orthopedic surgery [**8**].

The different incidence related to race or geographic area and the communicated familial cases may suggest the implication of a genetic mechanism. In familial cases, the disease seems to have a dominant transmission. One study on 122 patients with Zenker diverticulum communicates an incidence of 2% of familial cases, without a genetic modification that can be highlighted [**9**].

The patients with Zenker diverticulum frequently associate hiatal hernia. Some studies revealed the relationship between gastro-esophageal reflux disease and Zenker diverticulum, considering that the acid reflux can ascend theoretically to the pharyngeal level, determining mucosal injury at the Killian triangle and cricopharyngeal muscle hypertrophy. An incidence of up to 72% of gastro-eso-pharyngeal reflux was found in patients with Zenker diverticulum. The pathologic relationship between these clinical entities can only be speculated. Nevertheless, long term administration of proton pump inhibitors in patients with or without surgical treatment for Zenker diverticulum, can be justified [**10**].

Regarding the surgical indication, it is not yet well established, some authors state that the presence of the diverticular pouch is already a surgical indication, others establish the indication adapted to the case, according to symptoms, age, associated pathology and the presence of complications, attitude that we agree with. 

## Methods

Surgical statistics were published during 2001-2011, including a group of 11 patients (5 men), that underwent surgical treatment for Zenker diverticulum. The diagnosis was based on a clinical picture (severe dysphagia, regurgitations, weight loss) and investigations (barium passage, upper endoscopy).

Barium passage shows the diverticular pouch, giving details about size, localization, retention of contrast, position related to esophageal wall and esophageal motility.

Upper endoscopy evaluates the communication of the pouch with the esophageal lumen, the aspect of esophageal and intra diverticular mucosa, allowing tissue biopsy.

We found useful esophageal manometry, investigation that measures esophageal motility and we have used it recently, after the purchase of the equipment [**32**].

**Fig. 1:**
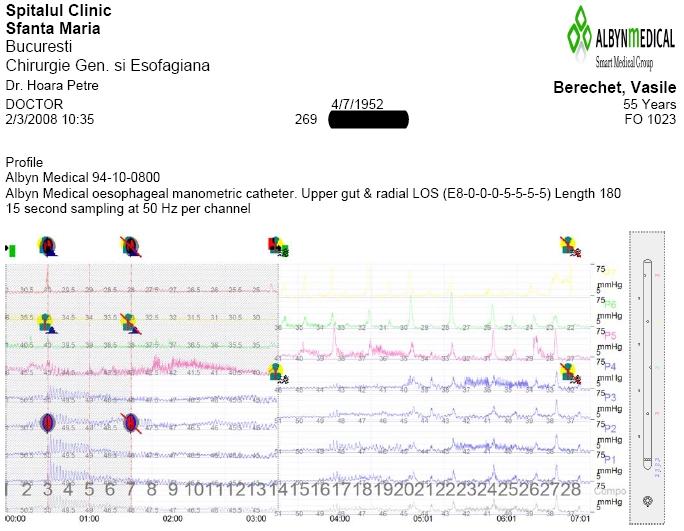
Esophageal manometry

All the patients included in the study underwent surgical treatment. Surgical indication was carefully evaluated, based especially on severe symptoms, resistant to conservative treatment.

We never used transoral approach, minimally invasive, promoted by some surgeons but dependent on expensive resources and difficult learning curve.

Surgical approach was left antero-lateral cervicotomy in all cases, regardless of the position of the diverticulum, due to left postero-lateral topography of the cervical esophagus related to trachea at this level, which makes its identification and isolation easier.

We have resected the diverticular pouch, an attitude that we consider good for improving dysphagia and preventing stasis complications and malignant transformation.

**Fig. 2:**
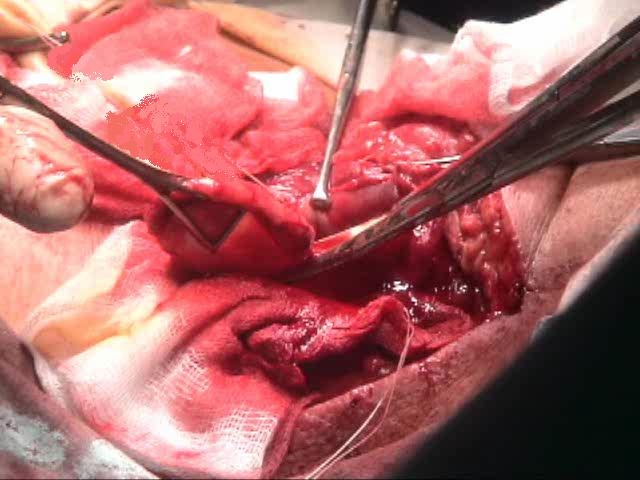
Resection of diverticular pouch

**Fig. 3:**
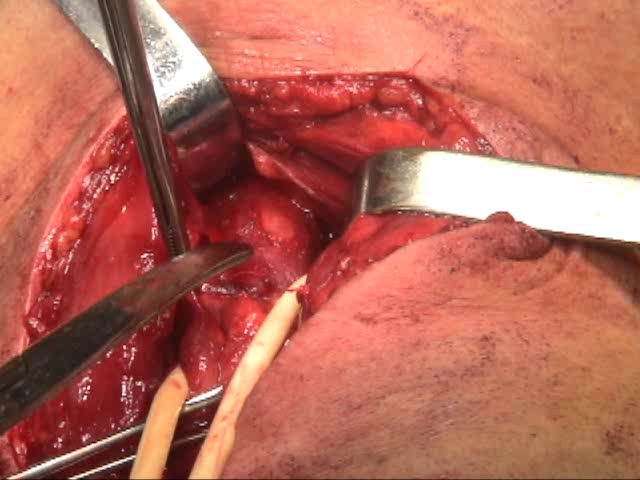
Esophageal myotomy

We have routinely performed esophageal myotomy, distal to the diverticular opening, regardless of the manometry findings or the intraoperative aspect of the muscular layer. We consider that myotomy is important for surgical treatment of esophageal diverticulum taking the etiology of the disease into account. 

## Results

There were 5 men and 6 women, mean age 54 years for men and 60 for women. The indication for surgical treatment was based on the severe dysphagia, which may cause in more than half of the patients, important weight loss.

Two cases with a cervical early postoperative hematoma have been recorded, a complication that required reintervention for evacuation and decompression, mandatory to prevent acute respiratory failure. In both cases, we could not identify the source of the bleeding, and there were no consequences in the evolution of the esophageal wound. The specific complication after surgery, esophageal leak, occurred early in 2 women patients. The esophageal closure was done manually with continuous double layer in both cases. In both cases, the evolution was favorable under conservative treatment that included partial opening of the cervical wound and extension of nothing by mouth period (NPO), but the hospital stay doubled. 

Early functional results were favorable in all the cases, with the disappearing of dysphagia. Long term results were hard to obtain due to short monitoring period in more than half of the patients. Especially in the two cases with esophageal leak, the follow-up time (3-4 years) was short and there were plenty of possibilities to obtain different results after the reevaluation of the patients.

## Discussion

Since 1796, when Ludlow had made the first description of the surgical treatment that was reported by Weeler in 1882, different therapies for Zenker diverticulum were imagined. Back in the 1912, Schmid communicated the diverticulopexy, with lower morbidity and mortality compared with the resection. In 1917, Mosher had introduced the technique that uses a rigid endoscope, with the section of the septum of the diverticulum, which was later improved by Dohlman in 1960 and Collard in 1993, as the performances of the endoscopes got better. In 1958, Harrison added myotomy in the surgical protocol of Zenker diverticulum [**11,12**]. 

Nowadays, therapeutic management of Zenker diverticulum implies 2 objectives, each one with specific technical details: the approach of the diverticular pouch and pharyngo-esophageal myotomy. Both objectives can be achieved by classic or minimal invasive through endoscopy.

Open surgery represents the most known approach of this pathology, especially in younger patients, due to long term favorable results communicated. The conservation of the diverticular pouch after diverticulopexy was for the first time described by Schmid in 1912, and made popular by Belsey and Skinner [**13,14**]. This should be done as high as possible, for evacuation. Diverticulopexy is realized with the sternocleidomastoidian muscle, prevertebral fascia, or exceptionally, the mastoid. This procedure does not require the opening of the esophageal mucosa, reducing the risk of developing fistula or stenosis, but includes myotomy. The technique permits the patient to resume oral feeding on the second postoperative day [**15**]. The opponents of this technique rise attention about the risk of abandoning a possible malignant lesion inside the pouch, on one side and on the other side, that the resection is not much more complicated than diverticulopexy [**16,17**].

**Fig. 4:**
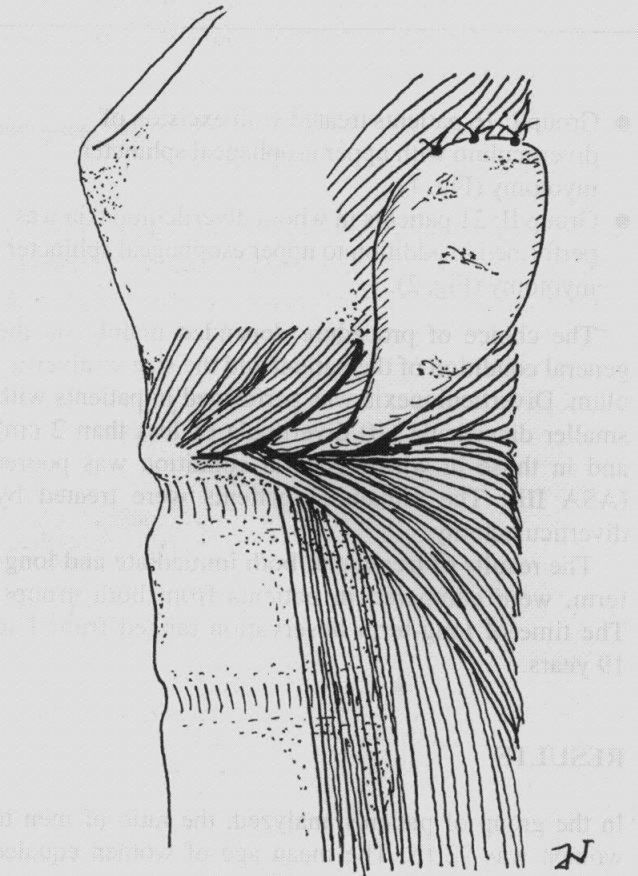
Diverticulopexy

**Fig. 5:**
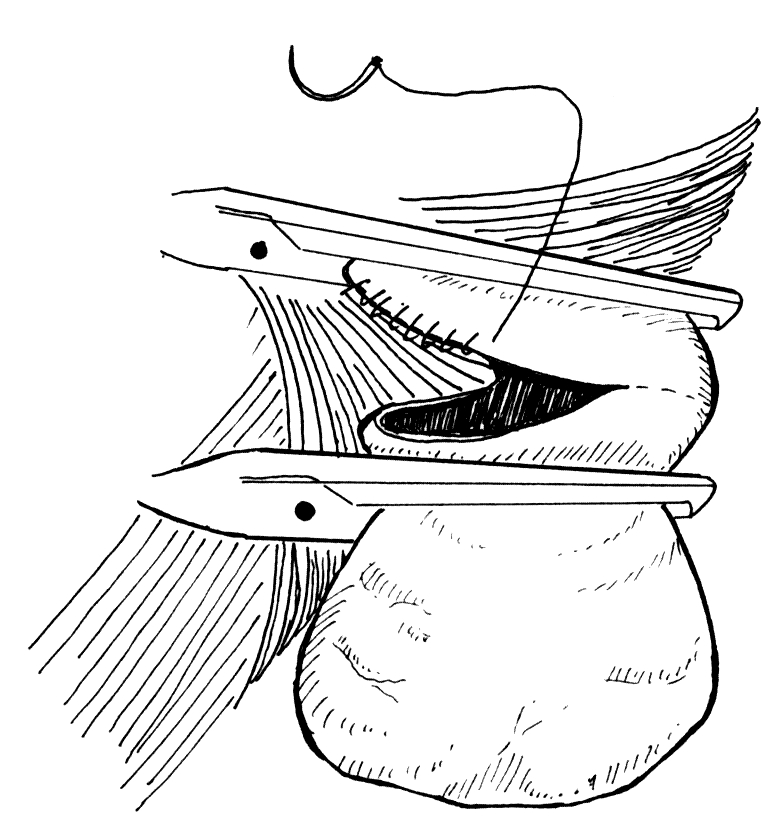
Diverticulectomy

Diverticulectomy is proved to be the most efficient method of alleviating dysphagia [**18**], compared with the simple myotomy or myotomy associated with diverticulopexy [**17**]. The technique implies the identification of the diverticulum, dissection of the adhesions with conjunctive tissue to the diverticular opening, which is just superior to the transversal fibers of cricopharyngeal muscle, resection of the pouch and restoration of the esophageal wall by manual suture in one or two layers. The section of the diverticulum using a linear stapler, followed by one layer of manual suture for safety, simplifies this approach.

If the surgical intervention is well done, the incidence of salivary fistula, specific complication of this operation, is minimal [**19**]. On the other hand, postoperative fistula at this level has a good prognosis under conservative treatment, being enough wide drainage by opening the cervical wound and NPO, with the healing of the leak in a couple of days. There are few cases in the literature with esophageal fistula that did not heal under conservative treatment, making iterative surgery necessary. In the second operation, it was found that there was an incomplete myotomy, the correction of the myotomy leading to the healing of the leak [**20**]. This kind of intraoperative findings support the etiologic theory of this disease, enhancing the importance of associating myotomy during surgery, whatever the attitude regarding the pouch is. This is considered the most important part of the surgical treatment by many authors [**19,21**]. Myotomy can be executed on the posterior midline, in order to avoid the relapsing that appears in case of lateral myotomy, before or after approaching the pouch, on 2-3 cm, and needs to cut the transversal fibers of crico-pharyngeal muscle, 1-2 cm from the fibers of inferior pharyngeal constrictor muscle [**22**] and 1 cm from the circular muscular layer of the esophagus.

Despite the fact that surgical morbidity decreased from 20-30%, where it was two decades ago, a lower percent in the present, mortality cannot be neglected, being around 1-2% [**23**]. All these have stimulated the development of alternative minimal invasive procedures.

In comparison with classic surgery [**24,25**], diverticulotomy with endo-stapler obtains favorable functional results (92-93%), similar with standard surgical approach (diverticulectomy and crico-pharyngeal muscle myotomy), instead of being minimally invasive. First attempts of endoscopic treatment of Zenker diverticulum, by cutting the muscular septum that separates the diverticular pouch from the esophageal lumen (diverticulotomy) belongs to Mosher, in 1917 [**26**]. Using the technique available at that time, the results were unacceptable, due to high incidence of mediastinal infectious complications. The idea was resumed by Dohlamn and Mattson in 1960 [**11**], by cutting the septum with the electrocautery, latter on using laser. Today, Dohlman’s technique was abandoned [**28**], because it is difficult to control the depth of the injury and there are too many risks (mediastinitis, fistula, hemorrhage, lesions of recurrent laryngeal nerve).

In 1993, Collard introduced diverticulotomy by using the endostapler, which decreased morbidity. The endoscopic procedure requires general anesthesia with tracheal intubation. The hypo pharynx is exposed using bivalve Weerda laryngoscope, which is positioned behind the endo-tracheal tube, by using an upper digestive endoscope, so that the two blades enter one in the esophageal lumen and the other in the diverticular pouch, exposing the separating septum. The septum is cut by using linear endostapler of 35 mm (EndoGIA), creating a common cavity, in the same time with the section of Upper esophageal sphincter [**25**]. The advantages compared with classic surgery are the absence of scar, diminished postoperative pain, early reestablishing of oral feeding and reduced length of hospital stay [**24**]. Specific complication is the perforation during the use of the stapler, the management of this complication implying conversion to open surgery and diverticulectomy [**31**].

## Conclusions

Although it has a “benign” pathology, esophageal diverticulum, requires complex surgical procedures that imply a significant morbidity rate.

The diagnosis is suspected based on clinical symptoms and easily confirmed by imaging studies available almost everywhere (barium passage and upper endoscopy).

Surgical indication must be carefully established, after thorough evaluation.

Esophageal manometry must be included in the investigations of these patients, on one hand for the assessment of dysphagia and on the other hand for optimization of indication and surgical technique.

The simple approach of the diverticular pouch through diverticulectomy or diverticulopexy is insufficient, the therapeutic protocol imposes the interception of etiologic mechanisms, esophageal myotomy distal to the diverticular opening being indispensable. This has to be done carefully, to the level of mucosa, regardless of the macroscopic look, often normal, of the muscular layer.

The simple myotomy as therapy, exposes the patient to specific risks given by the presence of diverticular pouch, including malignant transformation. After a long-term evolution, it is known that this complication is diagnosed almost exclusively at the pathologic examination of the diverticular resection specimen.
